# Strategy for Designing a Synthetic Tumor Vaccine: Multi-Component, Multivalency and Antigen Modification

**DOI:** 10.3390/vaccines2030549

**Published:** 2014-07-15

**Authors:** Zhi-Hua Huang, Zhan-Yi Sun, Yue Gao, Pu-Guang Chen, Yan-Fang Liu, Yong-Xiang Chen, Yan-Mei Li

**Affiliations:** Department of Chemistry, Key Laboratory of Bioorganic Phosphorus Chemistry & Chemical Biology (Ministry of Education), Tsinghua University, Beijing 100084, China; E-Mails: huangzh05@mails.tsinghua.edu.cn (Z.-H.H.); sunzhanyishiwo@126.com (Z.-Y.S.); marchisio8@126.com (Y.G.); woshicpg@126.com (P.-G.C.); lyfbenid@gmail.com (Y.-F.L.); chen-yx@mail.tsinghua.edu.cn (Y.-X.C.)

**Keywords:** tumor, vaccine, multi-component, multivalency, nano-size

## Abstract

Synthetic tumor vaccines have been proven to be promising for cancer immunotherapy. However, the limitation of the specificity and efficiency of the synthetic tumor vaccines need further improvements. To overcome these difficulties, additional tumor-associated targets need to be identified, and optimized structural designs of vaccines need to be elaborated. In this review, we summarized the main strategies pursued in the design of synthetic tumor vaccines, such as multi-component, multivalency, antigen modification and other possible ways to improve the efficiency of synthetic tumor vaccines.

## 1. Introduction

Tumor is one of the most dangerous diseases all over the world. Traditional therapies, such as surgery, chemotherapy and radiotherapy, are not always effective and may lead to serious side-effects. Therefore, immunotherapy is considered to be safer and more effective as a promising therapy [1].

In recent years, several kinds of immunotherapies have been focused on. Activated lymphocytes *in vitro* or antibodies are transported into patients to progress passive immunotherapy. Some immune-related molecules, such as IL-2, are used for activating the immune system as nonspecific immunostimulatory agents [2]. Synthetic tumor vaccines have also been developed to elicit an antigen-specific immune response to remove tumor tissue and to improve the quality of lives of patients [3].

## 2. The Challenges of Synthetic Tumor Vaccines

The first challenge of synthetic vaccines against tumor is to find the “right antigens” as targets [3]. Unlike tumors caused by oncogenic virus, such as human papillomavirus, which expresses specific virus-associated antigens, many kinds of tumor cells express self-antigens at higher levels. Therefore, a perfect tumor vaccine should elicit an immune response that could not only specifically recognize and kill the tumor cells, but also avoid the autoimmune responses. In some kinds of vaccines, extracts of tumor tissue or inactivated tumor cells are directly used as a kit of antigens [4].

Tumor antigens can be sorted into two parts, tumor-specific antigens, which only express on tumor cells, and tumor-associated antigens, which also express on the surface of normal cells at lower levels, such as glycoproteins and glycolipids [5,6]. Tumor-associated antigens are widely focused on in synthetic tumor vaccines, especially carbohydrates, glycoproteins and glycopeptides. Glycosylation is a kind of important modification associated with the processes of cell-cell interaction, immunological recognition and cell-vessel adhesion. Many specific changes of carbohydrate structures are observed during tumor progression. Therefore, glycoproteins and carbohydrates may be the potential targets of the design of tumor vaccines. There are many kinds of tumor-associated carbohydrate antigens and glycoprotein antigens. Carbohydrate antigens, such as Tn, TF, STn, GM2, Globo H, PsialA, Gb3, Le^y^, GM3, STn and ST, are widely focused on in the development of tumor vaccines [7,8]. The appearance of these antigens may be due to the upregulation or downregulation of different glycosylation-associated enzymes in tumor cells. MUC1 is a well-known tumor-associated glycoprotein antigen, which is overexpressed in many epithelial adenomas, such as colon, prostate, ovary and breast [9]. Moreover, overexpression of MUC1 on tumor cells is associated with tumor metastasis [10,11,12,13]. In tumor cells, MUC1 distributes on all surfaces of the cells. By contrast, MUC1 only expresses on the free surface of normal cells [14].

The second challenge of synthetic vaccines against tumor is to elicit a high immune response against a certain antigen. Tumor cells may escape the immunological surveillance and cause immunological tolerance during tumor progression. Because of immunological tolerance to tumor antigens, the immunogenicity of the self-antigen, especially small carbohydrates and glycopeptides in synthetic vaccines, is too weak to elicit a robust immune response. Therefore, besides the specificity, vaccines need to be powerful enough to break the tolerance and rebuild surveillance, which is important for long-term protection [15]. An adjuvant is also added to the vaccine dose to improve the immunogenicity of the antigen, providing vaccine delivery and an immunostimulator, such as Freund’s Adjuvant, MF59 emulsion, QS-21 and aluminum adjuvant [16]. Improving the immunogenicity of the antigen is a main aim of vaccine design.

## 3. Multi-Component Vaccine

### 3.1. Carrier Proteins in Vaccine Design

**Figure 1:**
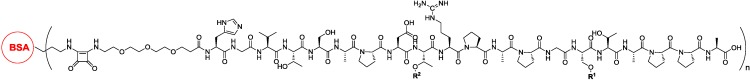
Li’s vaccine consisting of the glycopeptide antigen and the T-cell epitope [20].

Carrier proteins are widely used in commercially available combined vaccines. Carrier proteins, which have lots of antigens, are highly immunogenic. Therefore, the conjugation of antigens with carrier proteins, such as bovine serum albumin (BSA), keyhole limpet hemocyanin (KLH) and tetanus toxoid (TT), could improve immune response against the desired antigens. Kunz and co-workers conjugated TF-modified or STn-modified MUC1 glycopeptide to TT and found that these conjugates elicited a high-level immune response [17,18]. The IgG antibody elicited by these vaccines recognized not only tumor cells, but also tumor tissues from patients [19]. Li and co-workers conjugated a series of MUC1 glycopeptides to BSA as vaccine candidates and found that glycosylation at the Thr residue in the PDTRP domain played important roles in eliciting the immune response (Figure 1) [20]. However, carrier proteins often elicit a high-level of immune response against themselves, which is probably not useful for immunotherapy against the desired antigens.

### 3.2. T-Cell Epitope in Vaccine Design

Immunological research indicates some important processes in the pathway of immune responses. The B-cell epitope is recognized by the B-cell receptor (BCR) on the surface of B-lymphocytes to elicit a fast, low-level immune response to produce a low-affinity IgM antibody. However, activation of T-helper cells is necessary in the affinity maturation and class switch of antibodies. Therefore, the T-helper cell epitope is a potential component for improving the immune response by T-cell activation. Based on these previous studies, a more rational structure can be designed [21].

**Figure 2:**
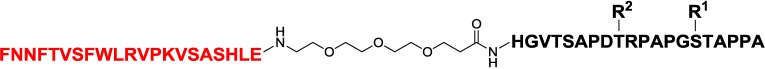
Li’s vaccine consisting of the glycopeptide antigen and the T-cell epitope [25].

Kunz and co-workers designed a two-component vaccine conjugating MUC1 glycopeptide with the T-cell epitope from carrier proteins using a flexible spacer [22,23]. The same strategy was used to synthesize vaccines consisting of different glycosylated MUC1 peptides. Vaccines of glycosylated MUC1 with STn on the Thr site in the PDTRP motif elicited the highest titer [24]. Li and co-workers synthesized vaccines consisting of 20-residue MUC1 glycopeptide and different T-cell epitopes from tetanus toxoid. Immunological evaluation demonstrated that the FNNFTVSFWLRVPKVSASHLE sequence could adjuvant the MUC1 glycopeptide to elicit a higher-level immune response without extra adjuvant (Figure 2) [25].

### 3.3. Toll-Like Receptor Agonist in Vaccine Design

The Toll-like receptor (TLR) in mammals is a kind of receptor that recognizes pathogen-associated molecular patterns (PAMPs) [26,27]. Agonist recognized by TLR may activate the pathway to regulate the expression of immune-related genes to activate the innate immune system and, consequently, the adaptive immune system [28,29,30]. Therefore, the TLR agonist can be used as an adjuvant in vaccine design. There are several kinds of TLRs that could recognize different agonists [28].

**Figure 3:**
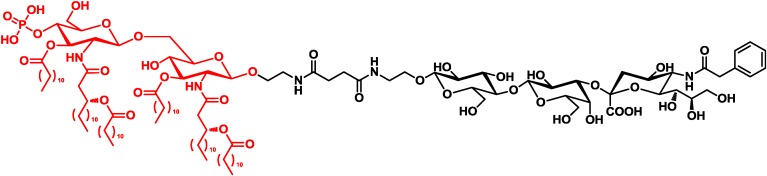
Guo’s vaccine consisting of the carbohydrate antigen and the TLR4 agonist [31].

Guo and co-workers designed a vaccine consisting of a GM3 carbohydrate antigen and monophosphoryl lipid A, an agonist of TLR4 from the cell wall of Gram-negative bacteria (Figure 3) [31,32].

**Figure 4:**
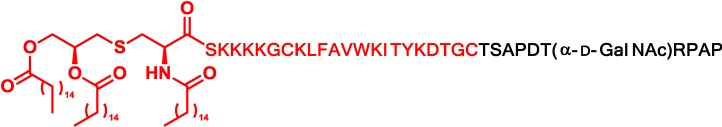
Boons’ vaccine consisting of the glycopeptide and the TLR2 agonist [34].

Lipopeptide from bacteria is a kind of TLR2 agonists that can be used as a building block in the synthesis of vaccines [33]. Boons and co-workers built an exciting structure consisting of a bacterial lipopeptide of Pam_3_CSKKKK, a short MUC1 glycopeptide as the B-cell epitope and a T-helper cell epitope of KLFAVWKITYKDT from poliovirus (Figure 4). The vaccine was immunized in liposome, eliciting specific and effective immune responses [34,35]. The uptake process of vaccines by the HEK293T cell line was obviously improved by the transfection of the TLR2 gene, which proved the functions of lipopeptide in the vaccine. Furthermore, the vaccine was proven to be capable of slowing the growth of xenograft tumor [36]. Payne and co-workers synthesized tumor vaccines containing per-glycosylated MUC1 glycopeptide and lipopeptide of Pam_3_CS with an efficient condensation reaction, and they demonstrated that different glycosylations affected the immune response [37]. Kunz and co-workers constructed vaccines containing Pam_3_CKKKK and MUC1 glycopeptide, which elicited a good immune response [38]. Li and co-workers developed the method of thioether ligation to ligate peptide and Pam_3_CKKKK, and they synthesized a series of two-component and three-component vaccines. These vaccines improved the immune response against antigens and killed tumor cells by complement-dependent cytotoxicity (CDC) [39]. Toth and co-workers developed an oligomer of lipoamino acid as the agonist of TLR2 and further synthesized vaccines, including carbohydrate antigens and this agonist [40].

Sucheck and co-workers designed multi-component vaccines in a different way [41,42]. When antigens are seized by antibodies, Fc domains of antibodies will interact with Fc receptors of phagocytes and induce the process of uptake, degradation and presentation of antigens. Therefore, Sucheck and co-workers first used a vaccine to elicit a high-level immune response against an unrelated antigen of l-rhamnose. This kind of antibodies can improve the presentation of l-rhamnose-containing three-component antigens by interactions between antibodies and carbohydrates and interactions between the Fc domains of antibodies and the Fc receptors of phagocytes.

## 4. Multivalency in Vaccine Design

The multi-component strategy is used to combine the components involved in different processes in the activation of the immune system. Moreover, multivalency is developed to achieve a cluster of antigens. It has been proven that cluster antigens could elicit a higher-level immune response than separated antigens, and antibodies recognized cluster antigens much better than separated ones [43,44,45]. Therefore, multivalency is another effective strategy to improve the efficiency of vaccines by combining several antigens in a single vaccine macromolecule [46]. Carrier proteins always bear several antigens in a single protein molecule, but it is hard to isolate a pure conjugate bearing a certain number of antigens.

**Figure 5:**
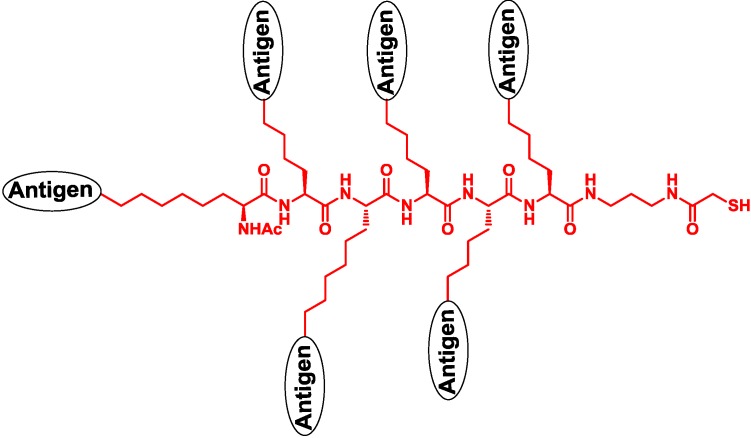
The structure of a linear multivalent vaccine.

Danishyfsky and co-workers designed a vaccine containing several kinds of carbohydrate antigens of Globo-H, STn, Tn, Lewis^y^ and TF in a linear backbone (Figure 5) [47,48,49,50]. In addition, a vaccine containing alternate Gb_3_ carbohydrate and an MUC5Ac T-helper cell epitope was designed to improve the immune response and specificity [51].

**Figure 6:**
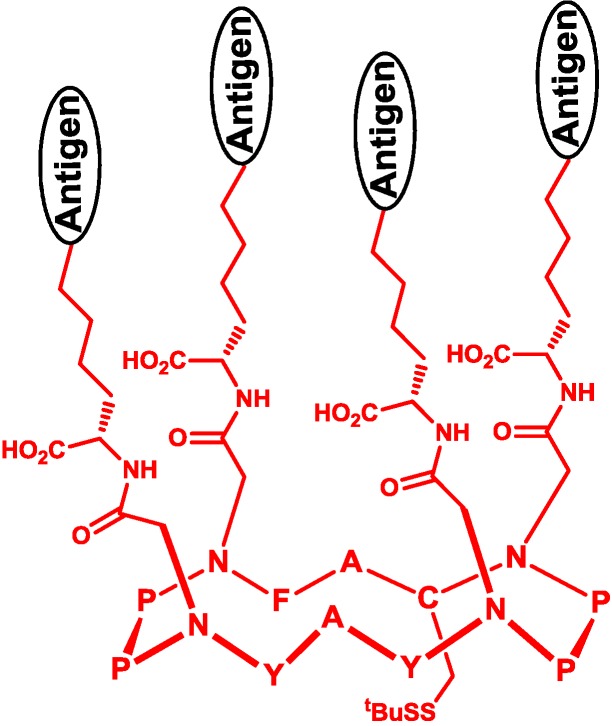
Multivalent template of a cyclopeptide.

Besides the linear backbone, cyclopeptide is also a useful template of multivalent vaccines, which can be formed by increasing the tendency of cyclization by proline [52] or the alternate sequence of l- and d-amino acid residues [53]. Danishyfsky and co-workers constructed a multivalent carbohydrate antigen on the cyclopeptide template and fixed the orientation of carbohydrate antigens by olefin metathesis (Figure 6) [54]. Dumy and co-workers designed a four-component vaccine consisting of multivalent carbohydrates on cyclopeptide, a T-helper cell epitope, a cytotoxic T-cell epitope and the TLR2 agonist [55]. This vaccine simultaneously elicited a humoral and cellular immune response, resulting in the suppression of the growth of MO5 xenograft tumor.

**Figure 7:**
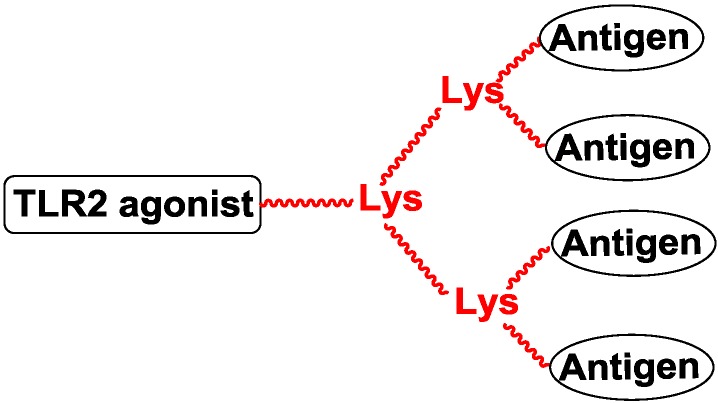
Multivalent template of polylysine.

Polylysine is also a template of multivalent structures. The number of amino group in the template could be doubled when adding another lysine to the template [56]. Li and co-workers designed four-valent vaccines by conjugating MUC1 peptide to a polylysine template with the reaction of azide-alkyne cycloaddition (Figure 7) [57,58].

**Figure 8:**
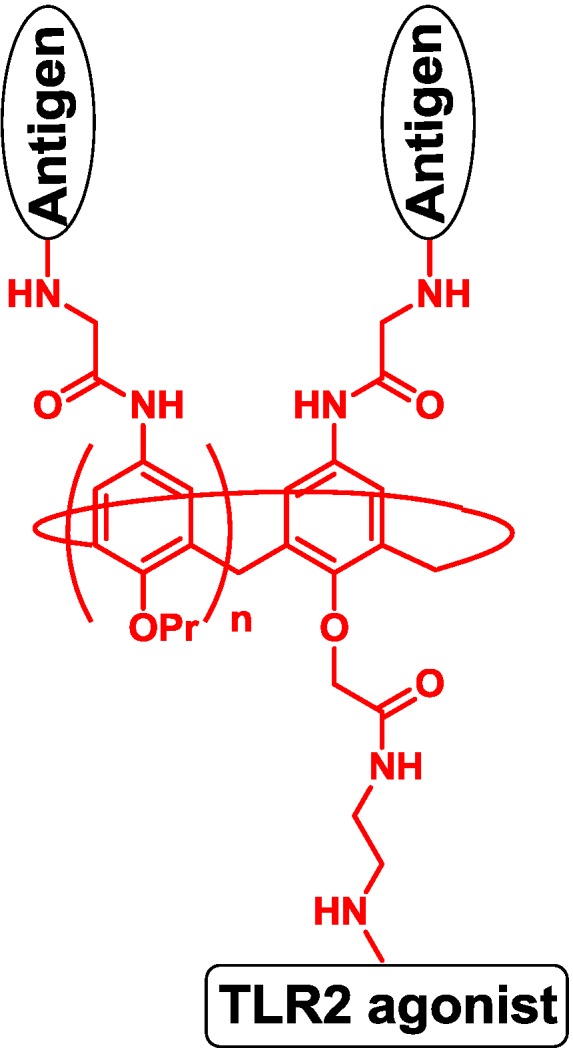
Multivalent template of calixarene.

Spadoro and co-workers designed four-valent and eight-valent vaccines using calixarene to present the PDTRP motif from MUC1. The TLR2 agonist was introduced to the template by the ether group, and the vaccine produced antibodies against the PDTRP motif (Figure 8) [59].

Polymers modified with functional groups also become a potential multivalent vaccine template. In this template, different monomers, various degrees of polymerization, block copolymerization and modifications can be employed to construct different vaccine structures. Kunz and co-workers synthesized poly(HPMA) and conjugated MUC1 glycopeptide, a T-helper cell epitope and lauric acid [60].

## 5. Antigen Modification

Vaccines with natural antigens often elicit a weak immune response, due to their structural characteristics or immunological tolerance. Therefore, besides the structural design of vaccines, strategies of antigen modification have been developed to enhance the immune response.

### 5.1. Modification in Vitro

A designed vaccine containing unnatural antigen is a powerful method to overcome the low immunogenicity or immune tolerance of natural antigens, which needs cross-recognition between antibodies elicited by unnatural antigens and natural antigens. Ye and co-workers synthesized kinds of modified STn antigens and found that fluorine modification enhanced the immunogenicity of the STn antigen [61]. The challenge of this strategy is to keep enough specificity of immune response against the desired antigens when analogs are used to elicit the immune response instead.

### 5.2. Modification in Vivo

Another interesting strategy for antigen modification is to modify antigens on the surface of tumor cells and then elicit the immune response to kill tumor cells with modified antigens. Schultz and co-workers designed a bifunctional molecule containing a 2,4-dinitrophenyl group and a specific ligand of a prostate-specific membrane antigen. This bifunctional molecule modified the prostate tumor cells with the highly immunogenic 2,4-dinitrophenyl group. A 2,4-dinitrophenyl vaccine was used to elicit the immune response to kill modified tumor cells [62]. Guo and co-workers modified the natural GM3 antigen by GM3NPhAc with a substrate of ManNPhAc by glycoengineering technology *in vivo*. Additionally, GM3NPhAc vaccines were applied to target modified tumor cells in mice models [63]. This strategy allows researchers to modify antigens with a simpler route or simpler substrates, improving “missiles” and “targets” in the same battle.

## 6. Conclusions and Perspective

Tumor vaccine is a promising strategy against tumor, which could elicit a systemic immune response to recognize and kill tumor cells and provide long-term specific protection for the body. Specificity and efficiency are the most critical problems to be overcome. Synthetic tumor vaccines showed their advantages in the design and optimization of tumor vaccines, and some effective strategies have been developed. However, immune response is a systemic result of complex interactions between molecules, cells, tissues and organs. It is too hard to forecast exactly the final effects of a certain vaccine. However, there are some principles that have been achieved, which could be used as references in vaccine design. T-cell activation is a key step for humoral immune response, so lack of T-cell epitope in vaccines may mainly cause the production of IgM antibody according to a T-cell-independent pathway [64]. T-cell response is regulated by the balance between co-stimulatory signals and immune check points, which apply contrary signals of stimulation and inhibition [65]. The differentiation of naive T-cells to different subtypes is regulated by cytokines and antigens, which affects the characteristics of immune response. The activation of the innate immune response could consequently activate the adaptive immune response, such as using TLR agonists. Tumor vaccines need to target different antigens or different immune-related pathways and elicit different types of immune responses, such as the humoral *versus* cellular response and activation of type 1 *versus* type 2 T-helper cell. A combination of different input immune-related components to optimize the output immune response is an important principle in vaccine design [16].

More than ten kinds of TLRs agonists were studied, such as imidazoquinoline, loxoribine, bropirimine for TLR7 and CpG DNA for TLR9. Many studies provided promising agonists for designing tumor vaccines [28,66]. Besides, it is worth noting that certain functional groups of agonists should stay free to remain their activities in the immune process [67]. Agonists of stimulatory receptors and antagonists of checkpoints, which have been proven to enhance the antitumor activity of other therapies, could be used as potential components of antitumor vaccines, such as inhibitors of PD1 and CTLA4 [68,69,70]. In addition, new templates could be used as multivalent tumor vaccines, such as carbohydrates with several hydroxyl groups [67,71,72]. Besides the design of single vaccine molecules that we discussed before, the size effect is also critical in the immune response by affecting the processes of antigen delivery, lasting antigen release, antigen presentation by antigen-presenting cells (APCs) and antigen cross-presentation [73]. Several studies have proven that vaccines with a relatively large size can have a stronger interaction with APCs, such as antigens of whole cells, virus-like particles and liposome delivery [74]. In fact, nanosized particles can be formed with traditional adjuvants, such as Freund’s adjuvant, aluminum adjuvant and liposome. Here, we just mentioned the nano-size behavior of vaccine molecules, but not with an additive [75,76,77,78,79,80,81]. Unlike carrier proteins, a proper nanosized system in tumor vaccines needs to elicit almost no immune response against itself. In the future, more attention should be paid to develop more rational and effective strategies for vaccine design to improve and strengthen the immune response and to modulate the characteristics of the immune response.
